# Time to Integrate to Nest Test Evaluation in a Mouse DSS-Colitis Model

**DOI:** 10.1371/journal.pone.0143824

**Published:** 2015-12-04

**Authors:** Christine Häger, Lydia M. Keubler, Svenja Biernot, Jana Dietrich, Stephanie Buchheister, Manuela Buettner, André Bleich

**Affiliations:** Institute of Laboratory Animal Science and Central Animal Facility, Hannover Medical School, Hannover, Germany; Cincinnati Children's Hospital Medical Center, University of Cincinnati College of Medicine, UNITED STATES

## Abstract

Severity assessment in laboratory animals is an important issue regarding the implementation of the 3R concept into biomedical research and pivotal in current EU regulations. In mouse models of inflammatory bowel disease severity assessment is usually undertaken by clinical scoring, especially by monitoring reduction of body weight. This requires daily observance and handling of each mouse, which is time consuming, stressful for the animal and necessitates an experienced observer. The time to integrate to nest test (TINT) is an easily applicable test detecting disturbed welfare by measuring the time interval mice need to integrate nesting material to an existing nest. Here, TINT was utilized to assess severity in a mouse DSS-colitis model. TINT results depended on the group size of mice maintained per cage with most consistent time intervals measured when co-housing 4 to 5 mice. Colitis was induced with 1% or 1.5% DSS in group-housed WT and *Cd14*-deficient mice. Higher clinical scores and loss of body weight were detected in 1.5% compared to 1% DSS treated mice. TINT time intervals showed no dose dependent differences. However, increased clinical scores, body weight reductions, and increased TINT time intervals were detected in *Cd14* ^*-/-*^ compared to WT mice revealing mouse strain related differences. Therefore, TINT is an easily applicable method for severity assessment in a mouse colitis model detecting CD14 related differences, but not dose dependent differences. As TINT revealed most consistent results in group-housed mice, we recommend utilization as an additional method substituting clinical monitoring of the individual mouse.

## Introduction

An important change regarding the legislation for the protection of laboratory animals has been the implementation of an exact severity assessment of all procedures undertaken on laboratory animals in the Directive 2010/63/EU on the protection of animals used for scientific purposes. According to Article 15 of the directive all procedures have to be classified into the categories “non-recovery”, “mild”, “moderate” and “severe” on a case-by-case basis. Furthermore, a prospective assessment and assignment of the classification of the severity of procedures has to be included in the application for the respective project authorization and subsequently the actual severity of the procedures performed has to be documented and reported accordingly (Article 38, 39 and 54 of the Directive 2010/63/EU). However, quantifiable parameters for the classification of severity into the postulated categories are still lacking. Therefore, a strong need for the exact determination of the degree of pain, suffering, distress or lasting harm experienced by the animal during the course of an experiment exists.

In mouse colitis models, which are valuable to study inflammatory bowel disease (IBD), it is common to utilize a clinical disease activity score for severity assessment [1, 2]. Clinical investigation of each individual mouse is time consuming and requires an experienced observer. The handling of the animal is obligatory and will cause additional stress to the animal.

IBD is a chronic, relapsing inflammation of the intestine with unknown etiology. The current perception is that this multifactorial disease stems from a genetically determined abnormal immune response against the normal intestinal flora leading to inflammation [3, 4]. To investigate the complex interaction of genetic and microbial factors and determine the exact components leading to the development of inflammation, animal models of IBD have been widely used [5, 6]. These experimental colitis models can be categorized with regard to the respective cause of inflammation into chemically induced models, spontaneously occurring models, genetically engineered models and cell/adoptive transfer models [7]. Among the chemically induced colitis models, the dextran sulphate sodium (DSS)-induced model is well established and widely used [8, 9]. Chemical induction of intestinal inflammation via DSS allows a fully controlled onset, duration and degree of severity of inflammation, thereby reducing variability within experimental groups. DSS treatment leads to an acute or chronic thyphlocolitis resembling UC in humans [10, 11]. Symptoms of DSS-colitis in mice therefore include weight loss and bloody diarrhea, consequently a daily welfare-assessment is obligatory.

As stated above, recognition of suffering or harm in laboratory animals is difficult due to a lack of quantifiable, validated and objective methods for measurement. Appropriate parameters for monitoring the development of diseases such as colitis and the health status of the animal are essential to define humane endpoints. We therefore aimed to evaluate whether the time-to-integrate-to-nest test (TINT) is suitable to detect disturbed animal welfare during the development of intestinal inflammation and whether it provides benefits over or in addition to a standard clinical scoring system. This easily performed test is based on the investigation of the strongly motivated nesting behavior of mice and detects disturbed animal welfare in consequence of painful surgical procedures [12, 13]. Nest building is a species-specific behavior in mice providing shelter from conspecifics, predators or direct light and which plays an important role for reproduction and thermoregulation [14, 15].

In this study, we additionally used the *Cd14*-deficient mouse. *Cd14* has been identified as a genetic modifier of experimental IBD whose expression level determines protection from disease and whose genetic deletion aggravates colitis [16, 17]. Comparing two different mouse strains (WT vs *Cd14* ^*-/-*^) treated with two different DSS doses (1% vs 1.5%) we analyzed whether the employed severity assessment strategy was sensitive enough to detect strain or DSS dose specific differences.

## Material and Methods

### Mice and Induction of DSS-Colitis

C57BL/6J (WT) and C57BL/6J.129S1-*Cd14* ^*tm1Smg*^ (*Cd14-deficient*; *Cd14* ^*-/-*^) mice were obtained from the Central Animal Facility (Hannover Medical School, Hannover, Germany). To induce colitis mice were exposed to 0% (control group), 1%, and 1.5% dextran sulphate sodium (DSS) in drinking water for 7 days. After 7 days the respective groups were euthanized by CO_2_ inhalation and subsequent cardiocentesis.

### Ethical Guidelines

This study was conducted in accordance with German law for animal protection and with the European Directive, 2010/63/EU. All experiments were approved and permitted by the Lower Saxony State Office for Consumer Protection and Food Safety (LAVES, license: 11/0499). Routine microbiologic monitoring according to recommendations of the Federation of European Laboratory Animal Science Associations did not reveal any evidence of infection with common murine pathogens [18, 19]. Mice were maintained in a room with controlled environment: 20–24°C; relative humidity 55±5%; 14:10 h light:dark cycle, 12–14 air changes hourly. Pelleted diet (Altromin1324, Lage, Germany) and autoclaved (135°C/60 min) distilled water were provided ad libitum. Mice were monitored for health and weight daily. During the study a low dose DSS concentration was used, thus mice showed only mild signs of severity. A body weight loss exceeding 20% of total body weight was defined as a humane endpoint.

### Clinical Scoring

Clinical assessment of animals was based on a previously published clinical score established to monitor severity in an experimental colitis model [20]. For each animal clinical parameters including stool consistency and general clinical condition were assessed and graded to quantify the severity of disease on a daily base (see Table 1). The loss of body weight was determined separately.

**Table 1:** Clinical colitis scoring.

Clinical parameters		Score
Stool consistency	Normal, soft, soft with blood	0–2
**General clinical parameters**		**Score**
Posture	Normal to hunched	0–2
Spontaneous behavior	Normal to no activity (without disturbing)	0–2
Provoked behavior	Normal to no activity (after disturbing)	0–2
Evaluation of the eyes	Clearness, openness	0–3
Evaluation of the fur	Cleanliness, gloss, smoothness	0–3
General appearance	Not, mildly, moderately, severely disturbed	0–3
**Total Score**		**17**

### TINT

TIN-testing was performed in accordance with recently published studies [12, 13]. Briefly, cages were bedded with hardwood shavings and nesting material (AsBe-wood GmbH, Buxtehude, Germany) was provided. A cotton-wool roll (AsBe-wood GmbH, Buxtehude, Germany) was cut into two halves and the surface was roughed so that the nesting material became fluffy. The cage top was opened and forceps were used to place nesting material in the corner of the cage opposite to the main nest site. Mice were observed for 10 min, and the time which was needed for integration was measured. All TINT observations in this study were made within 3 hours of light onset.

### Histology

The colon was prepared as a “Swiss roll” without being opened prior to rolling [21]. Colon samples were fixed in neutral buffered 4% formalin, processed routinely, embedded in paraffin, sectioned at 5–6 μm, and stained with hematoxylin and eosin. Histology slides were scored based on previously described colitis scores with histopathologic lesions graded separately for the proximal and distal colon [17, 22]. However, for further refinement the scoring system was adapted as follows [23]: Categories assessed were the presence of inflammatory cells (severity and maximum extent), the intestinal architecture (epithelial and mucosal), the extent of edema and the involved area. Each parameter was graded from 0 (no changes) to 4 (severe changes) as shown in Table 2 and calculated by adding the proximal and distal colon sections (maximum score of 46).

**Table 2:** Histology score.

Category/Parameter		Score
Presence of inflammatory cells	Severity	0 = no changes
		1 = mild
		2 = moderate
		3 = marked
		4 = severe
	Maximum extent	0 = no changes
		1 = mucosa, L. propria
		2 = + submucosa
		3 = + transmural, L. muscularis
		4 = breakthrough, peritonitis
Intestinal architecture	Epithelial	0 = no changes
		1 = focal erosions
		2 = marked erosions
		3 = several erosions
		4 = extended ulcerations
	Mucosal	0 = no changes
		1 = focal architecture loss, blunted crypts
		2 = moderate architecture loss, mucine retention
		3 = marked architecture loss, crypt necrosis
		4 = no architecture
Extent of edema		0 = no changes
		1 = edema in epithelial cells
		2 = edema in submucosa
		3 = edema in muscular layers
Area involved		0 = no changes
		1 = 25%
		2 = 50%
		3 = 75%
		4 = 100%

### Statistics

If not stated otherwise values are means ± standard error of the mean. All statistical analyses were performed using Graph-Pad Prism5 software (La Jolla, CA). For parametric data, t-tests and one-way analysis of variance (ANOVA) were carried out with Bonferroni´s multiple comparison test as post-hoc test. Two-way ANOVA was performed to compare data between mouse strains. *P*<0.05 was considered significant. * indicates *P*<0.05, ** indicates *P*<0.01, and *** indicates *P*< 0.001.

Untreated control groups consisted at least of 5 animals. DSS treatment groups of 7–11 animals. TINT group experiments were repeated 2 to 5 times with 4 to 5 animals each.

## Results

### Histology

**Fig 1:**
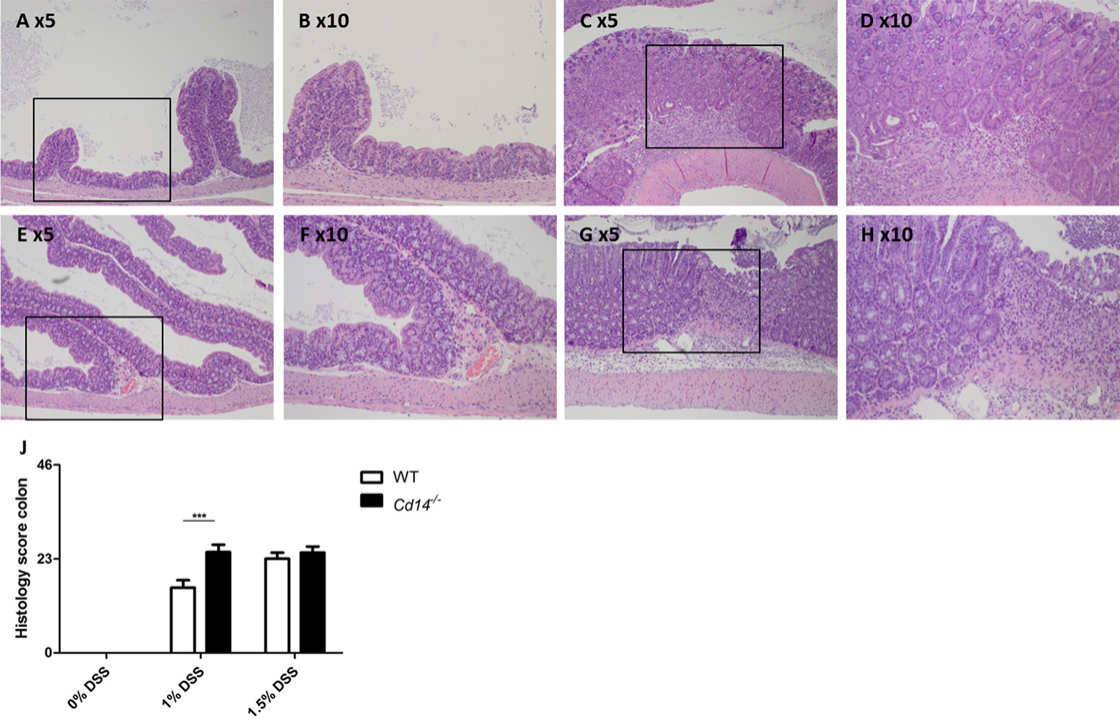
Intestinal inflammation induced by DSS-treatment. Hematoxylin and eosin staining of colon tissue obtained from (A-D) wild-type and (E-H) *Cd14*-deficient mice treated with 1% DSS for seven days (C-D and G-H, respectively). Untreated controls (A-B and E-F) did not show any signs of inflammation. Colitis was characterized by the presence of mixed cell infiltrates, hyperplasia, abnormal crypt architecture, edema and erosions (see boxed magnifications D and H). Original magnification 5x and 10x. Histological score quantifying the alterations observed in the colon (J).

Histologically, intestinal inflammation induced by DSS-treatment was characterized by cell infiltrates in the lamina propria and submucosa, abnormal crypt architecture, goblet cell depletion, edema and erosions as well as moderate ulcerations (see Fig 1C and 1D and 1G and 1H for an overview). No histological alterations were detected in the respective control groups treated with 0% DSS (see Fig 1A, 1B, 1E and 1F). However, all animals treated with DSS developed a profound colitis (Fig 1C and 1D and 1G and 1H). Furthermore, differences in inflammation were found between WT and *Cd14*-deficient mice. *Cd14*-deficient mice demonstrated significantly higher histology scores than WT mice when treated with 1% DSS (Fig 1J). After treatment with 1.5% DSS no significant differences were observed between WT and *Cd14*-deficient mice.

### Clinical Scoring

**Fig 2:**
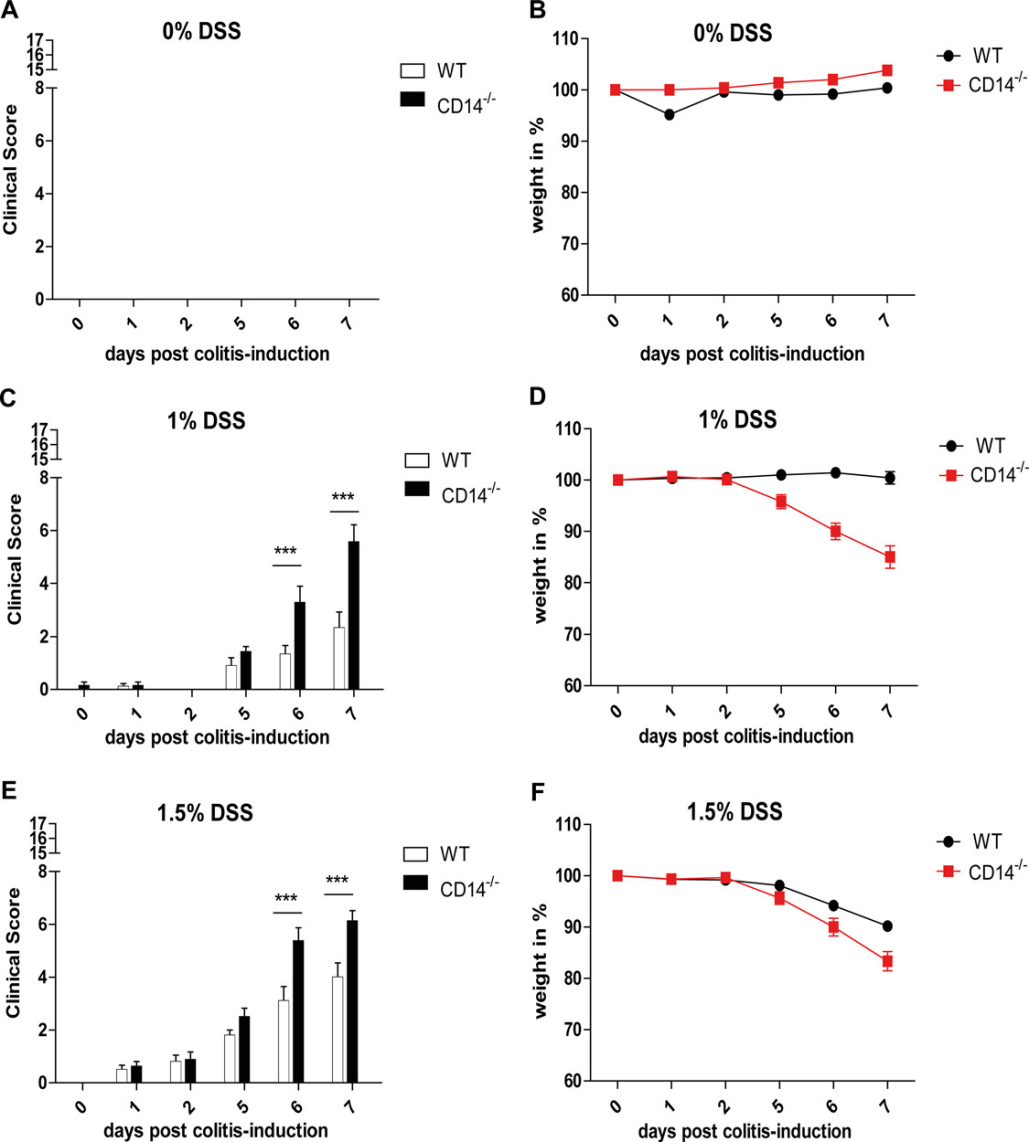
Clinical disease activity score after DSS treatment. Assessment of severity in controls (A, B) and DSS treated mice (1% DSS C, D and 1.5% DSS E, F) determined by an overall clinical disease activity score (A, C, E) and specifically by the change in body weight (B, D, F). Untreated controls exhibited low clinical scores (A) and a steady body weight (B). Mice treated with 1% (C, D) or 1.5% DSS (E, F) demonstrated increasing clinical scores and loss of body weight. *Cd14* ^*-/-*^ mice showed significantly higher clinical scores and a significantly higher reduction of body weight than WT mice.

For the assessment of severity in DSS treated and untreated wild type (WT) and *Cd14* ^*-/-*^ mice, the health status was investigated daily using a clinical score which included stool consistency, posture, behavior and the evaluation of eyes and fur (Table 1). Body weight development was evaluated separately. DSS treatment of WT as well as *Cd14* ^*-/-*^ mice led to an increased clinical score (especially in the parameters stool consistency and fur) and to a loss of body weight correlating with the increasing DSS concentration as well as the duration of treatment (S1 Fig). As shown in Fig 2A untreated WT and *Cd14* ^*-/-*^ mice showed no signs of severity reflected in a low clinical score and a steady body weight (Fig 2B). WT as well as *Cd14* ^*-/-*^ mice treated with 1% DSS (Fig 2C) demonstrated on day 5 post colitis-induction a slightly increased clinical score. In WT mice this slight increase continued to a score of approximately 2 on day 7 post colitis-induction. In contrast to this, clinical scoring of *Cd14* ^*-/-*^ mice revealed a significantly higher score of nearly 6 on day 7 post colitis-induction. These differences were underlined by a significantly higher loss of body weight in *Cd14* ^*-/-*^ mice than in WT mice (Fig 2D). Treatment with 1.5% DSS led to an earlier increase in the clinical score in both mouse strains, reaching a score of approximately 2 on day 2 post colitis-induction. Subsequent clinical scoring revealed a maximum score of 5 on day 7 post colitis-induction in WT mice but a significantly higher score of nearly 7 in *Cd14* ^*-/-*^ mice (Fig 2E). Correlating to this the reduction of body weight reached a maximum of ~10% on day 7 post colitis-induction in WT mice and ~15% in *Cd14* ^*-/-*^ mice (Fig 2F).

### Group Size Effect on TINT Results

**Fig 3:**
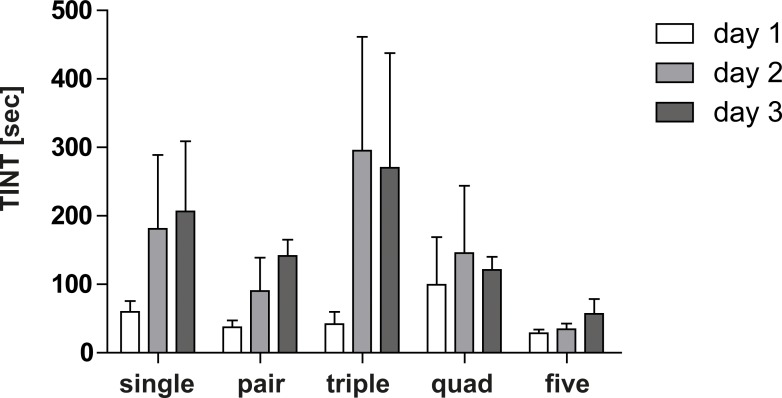
Group size effect on TINT reliance. TINT time intervals determined in untreated WT mice on three consecutive days. A group size of 4 to 5 mice per cage resulted in consistent time intervals.

For the evaluation of TINT in a mouse colitis model, initially a group size analysis was performed comparing cages with 1, 2, 3, 4 or 5 in-housed mice. TINT was conducted on three consecutive days with untreated WT mice determining time intervals for the integration of nest material. As shown in Fig 3, on the first day of observation TINT time intervals were similar in each group ranging between 20 and 60 seconds. Interestingly, on the second and third day of TINT performance, cages with 1, 2 or 3 in-housed mice demonstrated increased time intervals whereas cages with 4 or 5 in-housed mice showed only slight changes with more consistent data. Therefore, a group size of 4 or 5 mice per cage was chosen for subsequent colitis experiments and three days of training sessions were performed prior to DSS treatment.

### Implementation of TINT during Colitis Development

**Fig 4:**
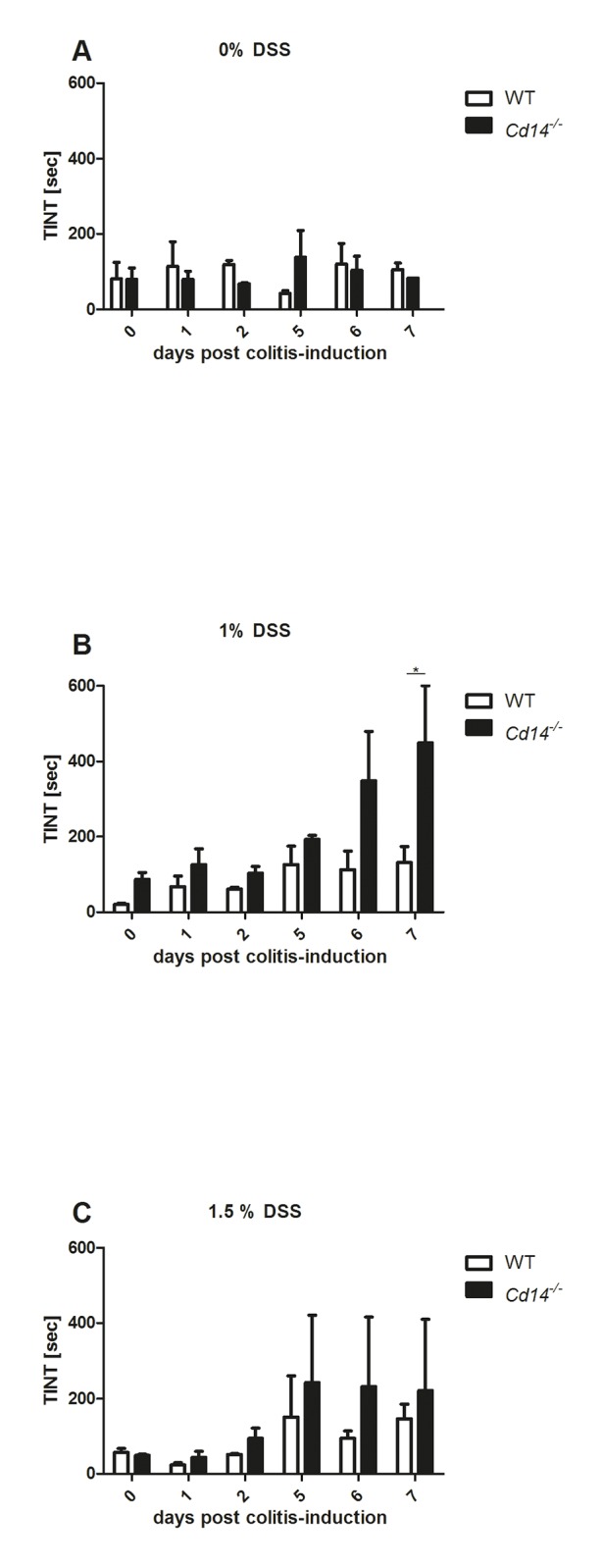
Colitis severity assessment by utilizing TINT. TINT time intervals determined in controls (A) and 1% (B) as well as 1.5% DSS treated mice (C). TINT time intervals were significantly increased in 1% DSS treated *Cd14* ^*-/-*^ mice compared to WT mice on day 7 post colitis-induction (B).

For the induction of acute intestinal inflammation WT and *Cd14* ^*-/-*^ mice were treated with 1% or 1.5% DSS for seven consecutive days. TINT was performed daily by adding nesting material to the respective cage and measuring the time mice needed for integration before each mouse was investigated with regard to clinical scoring. No differences were detected regarding the administered DSS dose, but mouse strain dependent differences were revealed (see Fig 4A–4C). TINT time intervals were elevated in *Cd14* ^*-/-*^ mice treated with 1% DSS beginning from day 5 post colitis-induction and increased slightly until the end of the experiments (Fig 4B). On day 7 post colitis-induction *Cd14* ^*-/-*^ mice demonstrated significantly higher TINT time intervals than the respective WT mice (Fig 4B). In general, corresponding WT mice showed no or only a minimal increase in TINT time intervals when compared to untreated control mice. In 1.5% DSS treated *Cd14* ^*-/-*^ mice elevated TINT time intervals were observed beginning from day 5 post colitis-induction sustaining on the same level until the end of the experiments (Fig 4C). 1.5% DSS treated WT mice responded with slightly increased TINT time intervals on day 5 post colitis-induction. However, compared to untreated controls these changes were not significant.

## Discussion

Aim of the present study was a comparative analysis of clinical scoring and observation of nesting behavior for severity assessment in a DSS mouse colitis model. We therefore used a clinical colitis score including the investigation of activity, general appearance and changes of body weight and the time-to-integrate-to-nest test (TINT) analyzing changes in strongly motivated nesting behavior. In line with previously published experiments control mice did not show any histological alterations, but all DSS treated animals developed colitis characterized by cell infiltrates, abnormal crypt architecture, goblet cell depletion, edema and erosions as well as moderate ulcerations. Furthermore, mouse strain dependent differences in colitis severity were detected. *Cd14* ^*-/-*^ mice, prone to respond to DSS treatment with an aggravated inflammation, demonstrated significantly higher histology scores when treated with 1% DSS than corresponding WT mice. Previously, CD14 was identified as a modifier gene for colitis with likely protective properties [16, 17]. These CD14 dependent differences in colitis severity were mirrored not only when applying the clinical score and measuring the body weight, but also when the TINT test was performed. Thus, CD14-deficiency results in a faster and stronger colitis development.

Another aspect of this study was the assessment of severity during development of intestinal inflammation. The assessment of severity has gained more importance since the new EU regulations (Directive 2010/EU/63) came into effect. Grading the level of discomfort an animal has to bear during experimental procedures is now strictly implemented in legislation and the severity of procedures has to be categorized into “non-recovery”, “mild”, “moderate” or “severe” on a case-by-case basis. Furthermore, a prospective severity assessment has to be included in the application for the respective project authorization and, finally, the actual severity an animal experienced during the procedures has to be documented in detail. However, the lack of validated methods and objective measurements as well as scientifically sound scales and references that enable to relate surveillance results to the degree of discomfort hinder such assessment at the moment. Therefore, it is obligatory to find new innovative strategies for severity assessment which are non- or minimal-invasive, straightforward and not time consuming as well as easy to perform. Furthermore, already existent methods like clinical scoring or behavioral studies have to be improved.

The clinical scoring system which was employed in this study was suitable to detect disturbed welfare in DSS treated mice. It was sensitive enough to detect strain-dependent differences between WT and *Cd14* ^*-/-*^ mice as well as dose-dependent differences between 1% and 1.5% DSS treated mice. Clinical scoring is a frequently used and widely accepted method which provides an identification of disturbed welfare conditions caused by colitis development. In general, clinical scoring in disease models like acute colitis or septic shock in laboratory animals includes assessment of posture, general appearance, weight loss, body temperature, activity and further parameter [20, 24]. Thus, this type of cage side observation is time consuming and the observer has to be well trained and experienced. In addition, the handling of the animal is obligatory and will cause additional stress to the animal.

The exact grading of severity requires a close-meshed scoring system and any quantifiable supplementary analysis would provide a benefit in this context. Among physiological or biochemical parameters, changes in animal behavior are very important indicators of disturbed animal welfare. Observance of strongly motivated behavior like nest construction in mice may be utilized to appropriately estimate stress or pain in laboratory animals. Postoperative pain or lesions of the brain lead to disturbed nesting behavior regarding complexity and motivation of construction [12–14, 25]. Other natural behavior patterns like burrowing and food hoarding are also suitable for detecting pain or stress in laboratory mice [26–29]. Examination of behavioral changes is commonly used in research focusing on psychiatric disorders. Anxiety- or depression-like behavior is generally analyzed using the open field test, the forced swim test, the elevated plus/zero/T maze or social interaction tests [30].

In the present study TINT was used for the analysis of nesting behavior as an indicator of disturbed animal welfare. TINT was supposed to be a fast and simple technique that can be used by non- experienced observers to identify pain in mice [12]. With the IBD model used in this study, we aimed at determining changes primarily not only due to pain but also other distress contributors affecting the animal in a multifactorial way. Because of the mild treatment regime used in these experiments the time measurement was modulated from a qualitative (10 min cut off) to a quantitative measurement (seconds) for a more precise assessment. As already described above elevated TINT time intervals were detected in DSS treated *Cd14* ^*-/-*^ mice compared to WT mice reliably detecting CD14 dependent differences in colitis severity. However, WT mice showed no significant increase in TINT time intervals during DSS treatment although the clinical scoring was mildly increased. Therefore, the strongly motivated nesting behavior seems to get disturbed only after exceeding a certain degree of distress (in this study the score of 6). This might discriminate mild from moderate distress induced by a certain procedure. Although both, TINT and the clinical score reliably demonstrated increases as early as day 5 post colitis-induction, TINT was not suitable to detect severity prior to the appearance of clinical signs. Thus, TINT was able to demonstrate severity of procedures but is limited in the identification of disturbed welfare in the individual mice. Furthermore, our data demonstrate for the first time that the group size has a considerable effect on TINT results. In our analysis single housing led to inconsistent time intervals and a high proportion of the animals failed to integrate the nesting material although they were untreated. Similar results were shown by Rock et al. [12]. Even in cages with 2 and 3 co-housed animals, data were very variable and inconsistent. Due to this finding, subsequent experiments were performed with 4 to 5 co-housed animals.

In conclusion, the results of the present study show that TINT is an easily applicable method for severity assessment in a mouse colitis model. The test was sensitive enough to detect CD14 related differences, although not dose dependent differences. As most consistent TINT results were gained in group-housed mice, we recommend utilization as an additional method substituting clinical monitoring of the individual mouse.

## Supporting Information

S1 Fig Clinical disease activity score and weight loss after DSS treatment. Assessment of severity in WT or *Cd14* ^*-/-*^ mice in untreated and DSS treated mice (1% DSS and 1.5% DSS) determined by an overall clinical disease activity score (A, C) and specifically by the change in body weight (B, D).
(TIF)
