# Supplementary figures and images for: Time to Integrate to Nest Test Evaluation in a Mouse DSS-Colitis Model
Source: PLoS One. 2015 Dec 4;10(12):e0143824. doi: 10.1371/journal.pone.0143824 (PMC4670219; doi:10.1371/journal.pone.0143824)

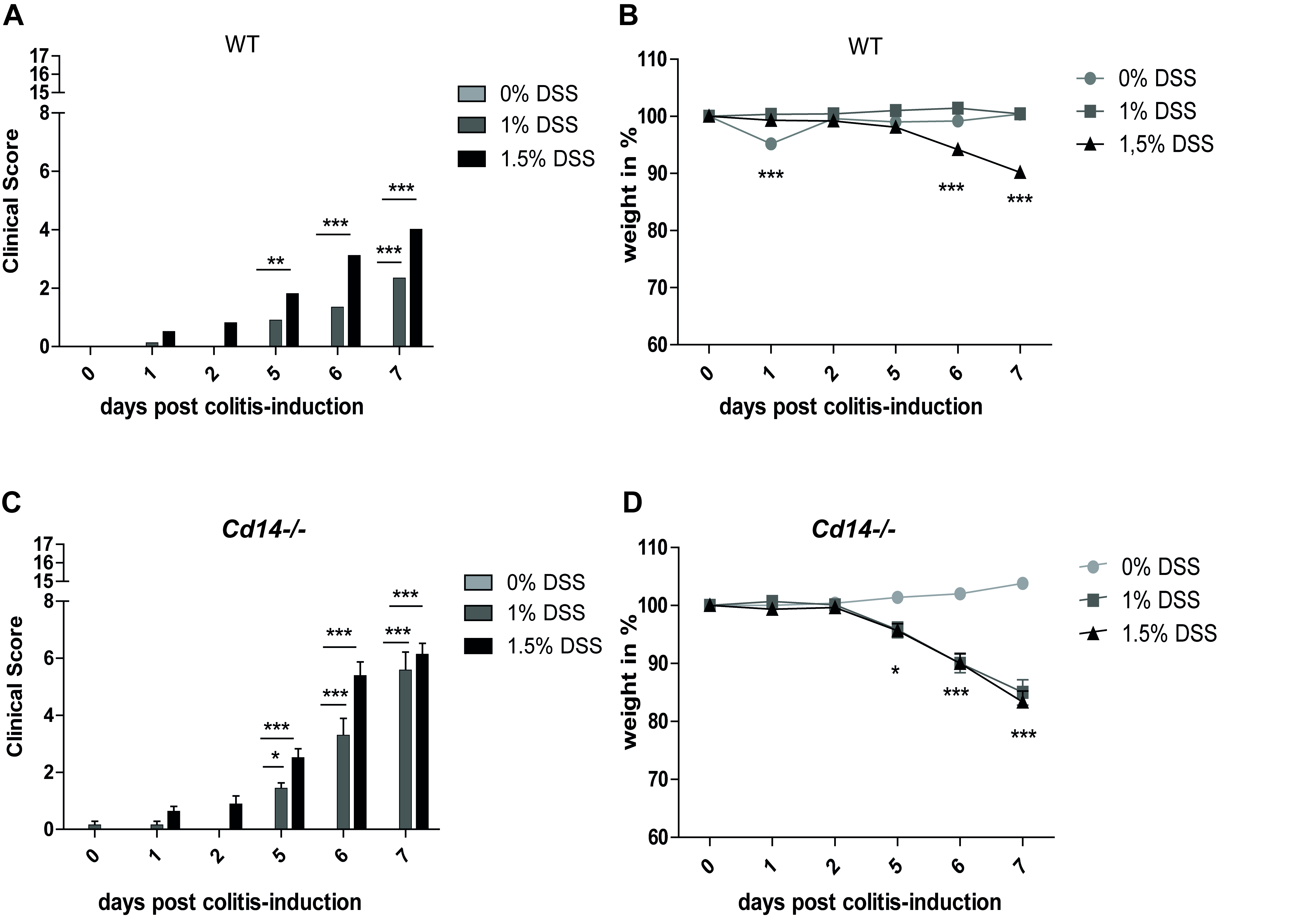

Supplement: S1 Fig — Assessment of severity in WT or Cd14 -/- mice in untreated and DSS treated mice (1% DSS and 1.5% DSS) determined by an overall clinical disease activity score (A, C) and specifically by the change in body weight (B, D). (TIF) [file pone.0143824.s001.tif]
